# Multi-modal body part segmentation of infants using deep learning

**DOI:** 10.1186/s12938-023-01092-0

**Published:** 2023-03-22

**Authors:** Florian Voss, Noah Brechmann, Simon Lyra, Jöran Rixen, Steffen Leonhardt, Christoph Hoog Antink

**Affiliations:** 1 Chair of Medical Information Technology, Helmholtz Institute for Biomedical Engineering, RWTH Aachen University, Aachen, Deutschland; 2 Fraunhofer Institute for Microelectronic Circuits and Systems, Duisburg, Germany; 3 KIS*MED (AI Systems in Medicine), Department of Electrical Engineering and Information Technology, Technische Universität Darmstadt, Darmstadt, Germany

**Keywords:** Deep learning, Neonatal intensive care, NICU, Semantic segmentation, Infrared thermography, Body part segmentation

## Abstract

**Background:**

Monitoring the body temperature of premature infants is vital, as it allows optimal temperature control and may provide early warning signs for severe diseases such as sepsis. Thermography may be a non-contact and wireless alternative to state-of-the-art, cable-based methods. For monitoring use in clinical practice, automatic segmentation of the different body regions is necessary due to the movement of the infant.

**Methods:**

This work presents and evaluates algorithms for automatic segmentation of infant body parts using deep learning methods. Based on a U-Net architecture, three neural networks were developed and compared. While the first two only used one imaging modality (visible light or thermography), the third applied a feature fusion of both. For training and evaluation, a dataset containing 600 visible light and 600 thermography images from 20 recordings of infants was created and manually labeled. In addition, we used transfer learning on publicly available datasets of adults in combination with data augmentation to improve the segmentation results.

**Results:**

Individual optimization of the three deep learning models revealed that transfer learning and data augmentation improved segmentation regardless of the imaging modality. The fusion model achieved the best results during the final evaluation with a mean Intersection-over-Union (mIoU) of 0.85, closely followed by the RGB model. Only the thermography model achieved a lower accuracy (mIoU of 0.75). The results of the individual classes showed that all body parts were well-segmented, only the accuracy on the torso is inferior since the models struggle when only small areas of the skin are visible.

**Conclusion:**

The presented multi-modal neural networks represent a new approach to the problem of infant body segmentation with limited available data. Robust results were obtained by applying feature fusion, cross-modality transfer learning and classical augmentation strategies.

## Introduction

As a preterm infant’s development inside the mother’s womb may not be fully completed, preterm birth can lead to serious health issues [1]. Globally, premature birth is the single most common cause of death among children below the age of five and accounts for over one million deaths per year [2]. If the infants survive, they are at increased risk of various physical and psychological illnesses, both short and long-term. These include, for example, respiratory, neurological, or cognitive conditions [3]. Since about 10% of children are born prematurely worldwide, this affects over 15 million infants each year. Nevertheless, the exact causes of premature births remain unknown. Therefore, there are few means for prevention, and the global number of premature births has even increased over the past years [4, 5].

On the other hand, neonatal intensive care and especially technical equipment in the Neonatal Intensive Care Unit (NICU) has continuously improved in the past decades [6]. Different technologies assist premature infants in their first weeks of life. They lie in incubators while respiration, heart rate and partial pressure of $\text {CO}_{2}$ and $\text {O}_{2}$ are monitored in order to detect potential health issues and counteract them [1]. In particular, for the infant’s survival, it is crucial to maintain a physiological body temperature [7]. At the same time, premature infants also lose heat to their surroundings faster than term newborns or adults due to their thinner skin and missing subcutaneous fat layer [8]. Therefore, the body temperature must be monitored continuously, and the heat supply in the incubators must be regulated precisely [9]. Additionally, it is essential to distinguish between peripheral and central body temperature. Hypothermia is first manifested by a drop in peripheral temperature, while the central temperature can still be kept constant by metabolic activity. An increased central–peripheral temperature difference may also be an early symptom of late-onset sepsis, one of the most common complications in neonatal care. Therefore, for early detection of cold stress or emerging sepsis, it is useful to continuously monitor the central–peripheral temperature difference [10].

Nowadays, the temperature of premature infants is commonly monitored by wired thermistors attached to the skin with strong adhesives. However, this can have adverse effects and lead to bacterial growth, skin damage, and irritations. Also, skin tears are possible during removal, which impair the skin’s barrier function [11]. In addition to the consequences of direct skin contact, the cables are a disturbing factor. Transport is made more difficult. and optimal positioning is not always possible due to the large number of cables in the incubator. Furthermore, it has been shown that the sight of cabling can cause parents to feel inhibited when interacting with their child [12]. For these reasons, a wireless and contactless alternative for temperature monitoring is desirable. Infrared thermography is a promising approach, where the temperature is obtained by measuring the emitted infrared radiation of the skin. However, to extract the temperature distribution of the neonate, it is necessary to automatically segment the skin of the regions-of-interest (ROI) since the location of these regions may vary due to movement.

Body part segmentation (Human Parsing) is a field of intensive research, with benchmarks having improved significantly over the past years [13–15]. However, the research on body parts segmentation of neonates, whose body proportions are significantly different from those of adults, is still not well examined. Chaichulee et al. [16], Dossso et al. [17] and Villaroel et al. [18] each segmented the skin of neonates in their work but did not distinguish between body parts. Zhang et al. [19] segmented body parts in videos of infants to detect possible neurological maldevelopment from the children’s movements. However, due to the small size of their dataset, their Neural Network was not able to robustly segment videos of children the network had never seen before.

Hoog Antink et al. [20] used a neural network for segmenting the body parts of neonates with a dataset of 643 manually annotated RGB and near infrared (NIR) images. They aimed to extract the infants’ heart rates without skin contact and achieved robust segmentation results, especially for the head, with a mean Intersection-over-Union of 0.82. However, these results were only obtained by pre-training the network on larger datasets for Human Parsing of adults. Training a network on 81 near infrared (NIR) images yielded significantly worse and unreliable results for the neonate segmentation.

Asano et al. combined a U-Net with a Generative Adversarial Network (GAN) and Self-Attention (SA) for body part segmentation in long-wave infrared (LWIR) images [21]. Opposite to Hoog Antink et al., they did not pre-train their neural network but only used a dataset containing 400 images of neonates.

In all the presented research, only one image modality was used for the body part segmentation. However, the fusion of different modalities has shown great success in other research fields. Farahnakian et al. [22] applied early, hybrid, and late fusion strategies for maritime vessel detection and compared the results to architectures only using images from visible light (RGB—red, green, blue) or LWIR. The fusion networks all achieved better results than the unimodal ones, with the RGB architectures being more accurate than LWIR networks. Early fusion approaches combine the information from two images on the pixel level before the images are processed further. This can, e.g., be done by concatenating an RGB and an LWIR image to form a four-channel image, which serves as the input of a neural network. Late fusion, in contrast, fuses the information on the decision or classification level. Separate neural networks thus process RGB and LWIR images, and the outputs are concatenated or merged afterwards [23]. The success of fusion strategies was also confirmed by Sun et al. [24]. They applied a feature fusion strategy using two encoders and one decoder to a semantic segmentation task fusing RGB and LWIR images of urban scenes.

To the best of our knowledge, there is no approach that directly compares the use of RGB images, LWIR images or a fusion of multiple image modalities for body part segmentation. In this work, we present a multi-modal approach for body part segmentation of infants. For this purpose, we solely used the information from RGB and an LWIR camera. With this data, we compared three approaches for the body part segmentation based on the robust and established U-Net architecture. First, the information from either the RGB or the LWIR camera was used for the segmentation by applying an encoder–decoder architecture. Additionally, we examined a hybrid fusion approach. The use and comparison of new transfer learning strategies for infant segmentation and especially segmentation in thermal images as well as a multi-modal approach for the problem of body part segmentation have so far not been reported in the literature, and are considered a novel contribution.

## Results

In the following, both unimodal and fusion networks were first optimized using the presented transfer learning and augmentation methods. Subsequently, the best models were trained incorporating all previous findings and evaluated on the left-out test data. Every training was set to run for 30 epochs for each fold with a batch size of 2, a learning rate of 0.0001, and a scheduled learning rate decay.

### Unimodal models

**Fig. 1:**
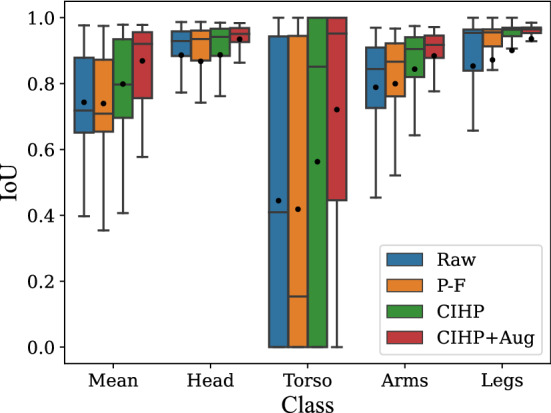
Box plot for cross-validation of the RGB model with (1) only the raw Chennai dataset (blue); (2) Pascal–Freiburg (P–F) pre-training (orange); (3) CIHP pre-training (green); (4) data augmentation and CIHP pre-training (red)

For the optimization of the RGB Model, we first examined the influence of transfer learning by pre-training on the Pascal–Freiburg dataset and the CIHP dataset. In a next step, the impact of data augmentation was investigated. The results of the different classes are depicted as box plots in Fig. 1.

As shown in Fig. 1, the models pre-trained on the CIHP dataset achieve a higher IoU for each class than the model that only uses the Chennai dataset for training. In contrast, the model pre-trained on the Pascal–Freiburg dataset even has worse results for the head and torso classes. In particular, the model pre-trained on the CIHP dataset achieves much better results on all body parts than the model pre-trained on the Pascal–Freiburg dataset. While the results for the head, arms, and legs only differ by a few percentage points, the CIHP dataset especially improves the segmentation of the torso. In addition, the standard deviation is reduced compared to the model with no pre-training. Due to the superior segmentation results, only the CIHP dataset is used for pre-training the models in the following. The model achieves an even better result when combining transfer learning and data augmentation, resulting in a mean IoU of 0.86. The augmentations improve all classes and decrease the std of the folds, resulting in an overall good segmentation of the infants when only using the RGB images. Only the torso is segmented suboptimal with an mIoU of 0.65.

**Fig. 2:**
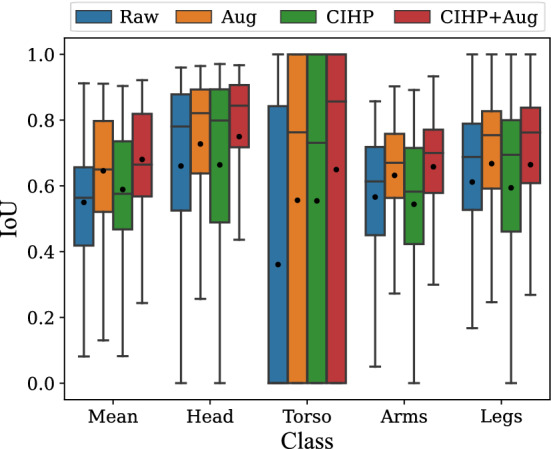
Box plot for cross-validation of the LWIR model with (1) only the raw Chennai dataset (blue); (2) data augmentation (orange); (3) CIHP pre-training (green); (4) data augmentation and CIHP pre-training (red)

Since the optimization of the RGB model has already shown that pre-training on the CIHP dataset yields the best results, transfer learning is only examined with a grayscale version of this dataset for the LWIR model. In total, four models were evaluated. Two models were solely trained on the Chennai dataset, with and without data augmentation. In addition, two models were pre-trained on the CIHP dataset, also with and without data augmentation. The results in Fig. 2 show the comparison of the classes and the standard deviation of the five folds.

Even though pre-training was performed only on a grayscale version of the CIHP dataset, the transfer learning improved the IoU by almost three percentage points compared to just training on the LWIR images of the Chennai dataset. Data augmentation further improves the training, resulting in an mIoU of 0.67. When comparing the training with augmentation and no pre-training, it is noticeable that the transfer learning even resulted in an improvement of 5 percentage points (from 0.62 to 0.67). Similar to the RGB model, inferior results were obtained for torso segmentation (IoU of 0.56). The remaining classes were segmented relatively well, with an IoU between 0.66 and 0.76.

### Fusion model

**Fig. 3:**
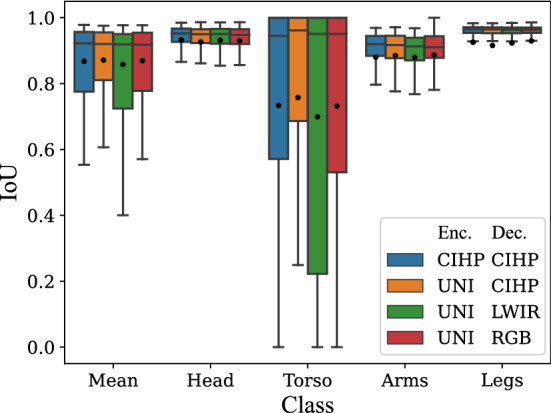
Box plot for cross-validation of fusion model with data augmentation and different fusion pre-training strategies for encoder (Enc.) and decoder (Dec.) (UNI = weights taken from unimodal RGB and LWIR encoders and used for the fusion model)

In the following, the modality fusion model is evaluated. Since data augmentation showed great success in the unimodal networks, it was used for all training of the fusion model. To optimize the combination of RGB and LWIR images, the four different pre-training strategies are evaluated. The results in Fig. 3 show the comparison of the classes with box plots.

As can be seen in Fig. 3, the model pre-trained only on the CIHP dataset combined with the established data augmentation methods already achieves good results with an mIoU of 0.86. Using the decoder weights of the unimodal networks does not further improve the segmentation results. In this case, the torso is detected even worse compared to only pre-training on the CIHP dataset. The best segmentation was achieved using the unimodal encoder weights and the pre-trained CIHP decoder weights. Here, the torso was segmented well with an IoU of 0.74, leading to an IoU improvement of 4 percentage points compared to only pre-training on the CIHP dataset. In total, the fusion of LWIR and RGB images as input for the neural network resulted in a mean IoU of 0.87.

### Evaluation on test set

**Fig. 4:**
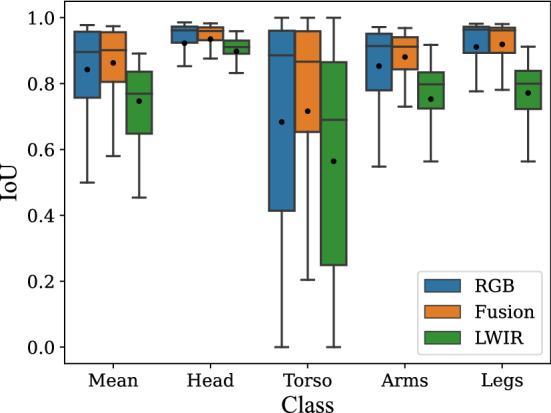
Box plot for the evaluation of the three different networks on the test set (with pre-training and augmentations), mean is marked with a dot

Combining the findings from the previous sections, the three final models were trained on all training data and evaluated on the test set. The LWIR and RGB models were first pre-trained on the CIHP dataset (the grayscaled CIHP dataset, respectively) and data augmentation was applied. The encoder weights of both unimodal models were then used as a baseline for the final evaluation of the fusion model. The decoder weights were taken from pre-training on the CIHP dataset. The final evaluation results for the three networks can be seen in Fig. 4, depicted as box plots.

**Fig. 5:**
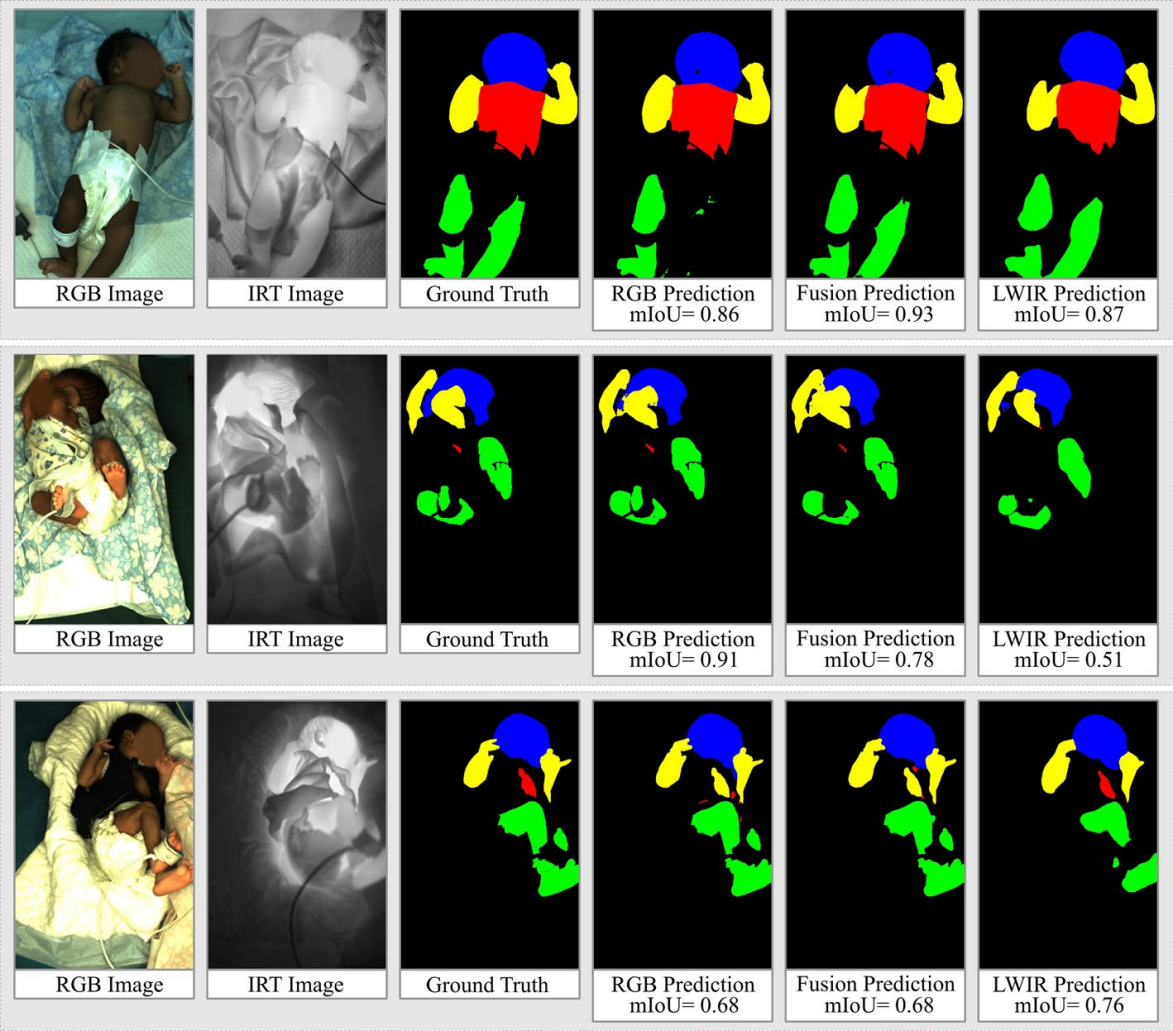
Qualitative results of the three models evaluated on the test set

We note that the fusion model achieves the best result with a mean IoU of 0.87, closely followed by the RGB model with an mIoU of 0.84. However, the LWIR model achieved worse segmentation results, with an mIoU of 0.75. Looking at the individual classes, it is evident that the head was segmented best by all three networks, followed by arms and legs. The torso was detected with the lowest accuracy, similar to the cross-validation Here, significant outliers were present, resulting in a big difference between median IoU and mean IoU. For example, the difference between the fusion model is 0.90 (Median IoU) and 0.69 (Mean IoU). In total, the fusion model achieved similar or better results than the RGB model for all classes, with the LWIR model being between 3 and 15 percentage points worse. Qualitative examples of the segmentation results are shown in Fig. 5.

The top row shows an example for a superior result of the fusion model. While the RGB model does not detect the left leg and the LWIR model has problems with the segmentation of the torso, the fusion model achieves good results in segmenting both the legs and the torso. An example of the superior results of the RGB model can be seen in the middle row of Fig. 5. It is the only one of the three models that segments most of the head and the arms correctly.

Lastly, the bottom row provides an example where the LWIR model produced the best results. In contrast to the RGB and fusion network, the LWIR network segments the torso skin, providing a good result.

## Discussion

### Hyperparameter tuning

The cross-validation from “Unimodal models” section shows that the transfer learning results are superior to the networks only trained on the Chennai dataset. Moreover, the CIHP dataset is found to be better suited for our application than the Pascal–Freiburg dataset that was previously used by Hoog Antink et al. [20]. Even though the legs are only visible in about every fourth image of the CIHP dataset, this disadvantage is compensated by the ten times larger size of the CIHP dataset compared to the Pascal–Freiburg dataset. In fact, the corresponding segmentation results on the legs were better than that of the arms and are still better than the results achieved by the model pre-trained on the Pascal–Freiburg dataset.

In contrast to Asano et al. [21] we also used transfer learning when training the neural network on LWIR images. Even though, in this case, transfer learning was performed with a grayscaled version of the CIHP Dataset, it also improved the results significantly. This indicates that for semantic segmentation and body part segmentation, respectively, cross-modality transfer learning is possible and can help to achieve better results. Nevertheless, it would be desirable to have a larger LWIR image dataset of infants/adults.

In addition to transfer learning, the used data augmentation strategy also improved the segmentation results for every trained neural network, ranging between 5 and 10 percentage points depending on the model. In contrast, the results from Hoog Antink et al. [20] even deteriorated slightly when data augmentation was applied. They assumed that their network might be able to generalize better when augmentations are used, but this may not be necessary to achieve good results on the validation data from their relatively small dataset with a total of 563 RGB images.

Similarly, the Chennai dataset is only slightly bigger than the dataset used by Hoog Antink et al., with variations of color, brightness, camera position, etc., not being vast. This may introduce a bias to, e.g., the skin color. We conducted additional experiments (not shown in “Results” section) to determine the influence of data augmentations such as color/brightness/saturation variations on the skin. This resulted in the same accuracy than without any augmentations, which indicates a bias in terms of skin color in the Chennai dataset. However, data augmentations such as color/brightness variations are necessary when applying the neural network in real-world scenarios or to new data with larger variations in skin color, brightness, etc. This needs to be evaluated with more data in future work. Furthermore, we used the grayscale RGB images of the CIHP dataset to perform transfer learning on the LWIR model. Since there are no large thermography databases with labeled body parts, this was the only feasible option, which improved the results, even though the modalities are different. In the future, it would be beneficial to generate more thermography data or even artificial image data to enhance transfer learning.

In this work, we chose a DenseNet121 as the backbone for the neural network. However, a DenseNet is often less memory/speed efficient as other network structures. Since this work is the basis for non-contact temperature measurement, the interference time is not as important as in other applications. Nevertheless, new backbones such as Repvgg by Ding et al. [25] should be investigated in the future for segmentation tasks. This can improve the interference time and enable other applications such as camera based vital sign measurement.

### Image fusion

The results from “Evaluation on test set” section are similar to Farahnakian et al. [22]. The fusion model achieves the best results compared to unimodal models. In addition, the RGB models perform better than the LWIR models.

In contrast to Farahnakian et al., our fusion model achieved only slightly better results than the unimodal ones, with some classes even being segmented worse. This is counterintuitive, since the fusion models can rely on more information to solve the task. Farahnakian et al. [22] did, in fact, find that the fusion models all performed at least as good or better than the unimodal ones. However, they worked on detecting maritime vessels using RGB and LWIR images with a distance between targets and cameras of several tens or hundreds of meters. In this scenario, the differences in perspectives due to the use of two separate cameras are less relevant than when the cameras are only a maximum of two meters away from the objects to be segmented, as is the case here. Similarly, Ha et al. [26], Li et al. [27], Sun et al. [24], Wang et al. [28] and Zhang et al. [23] all fused multi-modal images but used images with targets being significantly further away from the cameras than the babies in the Chennai dataset (e.g., landscapes, ship traffic, satellite images, urban scenes, etc.). Ha et al. [26] and Sun et al. [24] further used images taken by a single camera that can record both RGB and thermal images simultaneously. Even though the improvement due to image fusion is marginal, it is still reasonable to use both modalities for segmentation, since the results will be used for the application of skin temperature measurement of infants. Hence, a thermal imaging camera is required in the measurement setup anyway.

Additionally, in contrast to other multi-modal studies, our LWIR images often have a lower contrast between the segmented object and the background, which may complicate the segmentation on this modality.

As described in “Image registration” section, manually transforming RGB and Ground Truth images does not provide a perfect registration. This may explain why the fusion model evaluated in this work partly performs worse than the unimodal networks. Whether better image transformations can further improve the segmentation results of the fusion models needs to be analyzed in future work. The transformation matrices can, for instance, be calculated automatically instead of manually. Calibrating the two cameras using calibration objects, however, must be done before the images of the babies are recorded and cannot be done retrospectively.

We also tested out different fusion strategies in the form of concatenating the extracted feature of the different modalities. However, this resulted in the same IoU of 0.87 during hyperparameter optimization. Since the operation of concatenating adds six million more weights to the fusion model, we chose the add operation instead. We also tested other fusion methods (early fusion and late fusion), but these also resulted in worse results.

### Class prediction

The evaluation of all three models indicated good segmentation results for all classes, with the head being segmented best. However, all networks show that the worst results are consistently achieved for the torso. This may be due to the fact that clothes covered the torso for most of the infants in the Chennai dataset. In Table 1, the average percentage of pixels of each class from the whole image can be seen.

**Table 1:** Classwise percentage of pixels for the Chennai dataset

Class	Background	Head	Torso	Arms	Legs
Percentage	83.4%	4.6%	1.8%	3.6%	6.5%

On average, the torso skin accounts for only 1.8% of the total image. In fact, the torso skin was only well visible for six recorded infants. In the other images, the torso was either entirely covered by clothes or only small parts of the torso were visible. On these infants, the networks struggle to achieve good segmentation results.

**Fig. 6:**
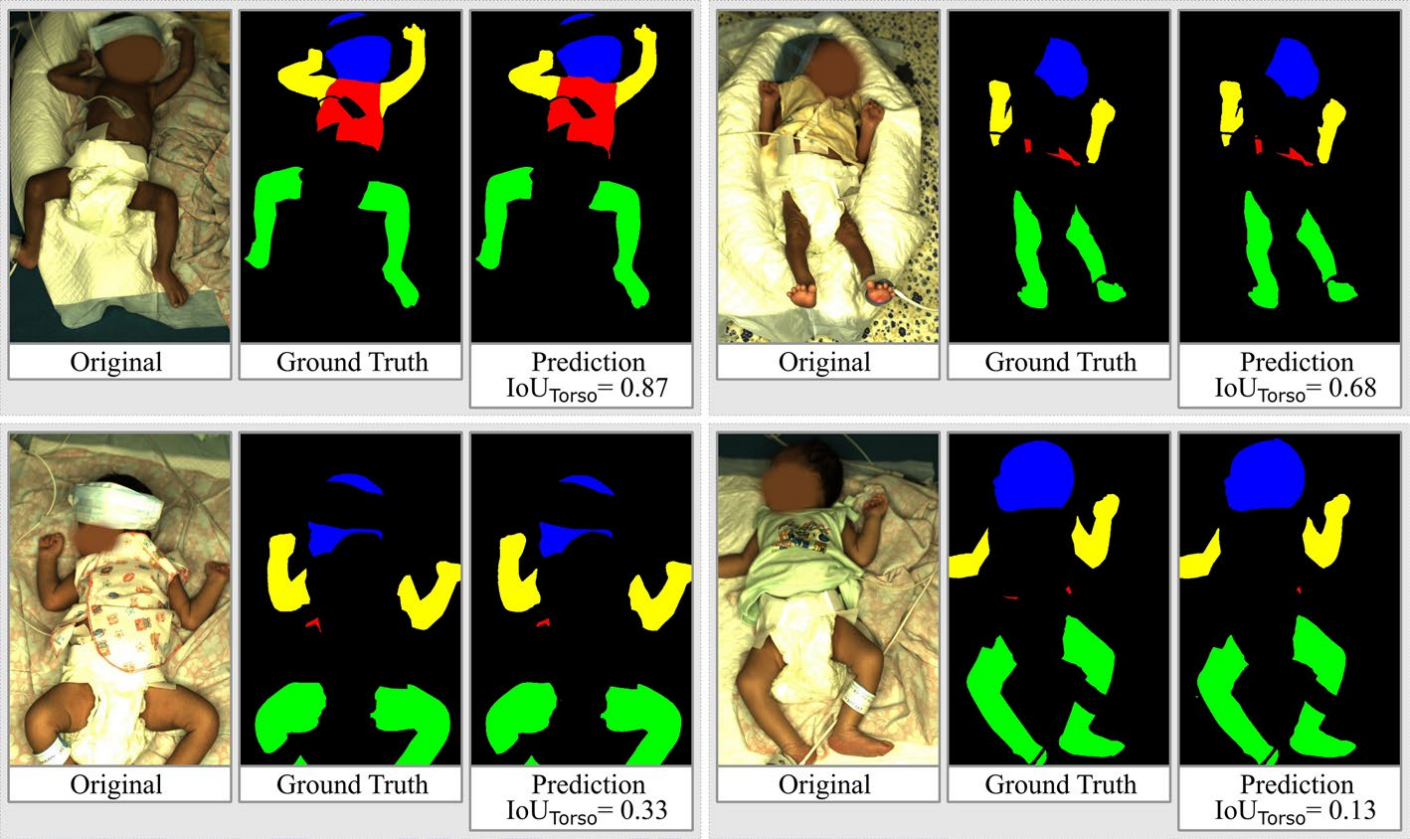
Examples of images and respective ground truth/prediction for different qualities of torso segmentation

Figure 6 illustrates that the IoU on the torso decreases as the size of the visible skin area decreases. However, in the context of temperature measurement, a certain size of skin area is necessary to obtain a reliable result. That is why the temperature cannot be taken from skin patches as small as the torso skin visible in some parts of the Chennai Dataset anyway. Therefore, it is reasonable to define a threshold for the size of visible skin areas from which temperatures are extracted. When this strategy is employed, suboptimal segmentation results on the torso become less relevant.

## Conclusion

In this work, a deep learning model was developed that automatically segments the individual body parts of infants. For this purpose, we compared three neural networks based on the widely known U-Net architecture that have either RGB or LWIR images as input or use a fusion of both image modalities. Furthermore, a dataset of infants comprising 600 RGB and 600 corresponding LWIR images was manually annotated and used for training and evaluation. Individual optimization of the three models showed that transfer learning with the CIHP dataset and data augmentation significantly improved the segmentation results, regardless of the imaging modality used as input. Surprisingly, cross-modality transfer learning also showed a significant improvement in the results of the LWIR model.

Combining all previous findings, final RGB, LWIR, and fusion models were evaluated on a separate test set. The fusion model produced the best segmentation results, with the RGB model providing comparable results. Only the LWIR model achieved a lower accuracy. The fusion model especially performed better on frames in which the contrast between skin and clothes or background was low in the RGB but high in the LWIR images. This suggests that the fusion model may be superior to the RGB model, especially when the illumination is turned off (e.g., at night). However, there is no image data available for this case yet, even though the night makes up a substantial part of a 24-h monitoring cycle. Neural networks trained on the Chennai dataset are hence biased, and especially the RGB model is overfitted to good illumination. Therefore, a more extensive dataset needs to be created to further evaluate and improve the deep learning models developed in this work.

When creating such a dataset, particular attention should be paid to some aspects. First, the dataset should contain images taken at night. Second, compared to the Chennai dataset, a wider range of skin colors, body positions (especially prone) and clothing of the infants should be covered. Third, the transformation matrices used to transform the RGB and ground truth images to compensate for the different camera perspectives should be calculated automatically (e.g., using calibration objects). Overall, the results of this work demonstrate new methods for improving infant segmentation results and may help provide more insight into the problems and challenges of infant body part segmentation, especially in light of the still limited amount of data available for this application.

## Materials and methods

### Dataset

The dataset used in this work was recorded by Lyra et al. [29] in the NICU of Saveetha Medical College & Hospital in Chennai, India. The study was approved by the institutional ethics committee of Saveetha University (SMC/IEC/2018/03/067). The study included 19 stable patients with gestational age at birth ranging from 29 to 40 weeks. Since one patient was recorded twice, there are in total 20 different recordings. The weight varied from 1500 to 3010 g, and the age from 37 h to 56 days post-birth.

RGB images were obtained using a Grasshopper 3 GS3-U3-23S6C-C (FLIR, USA) with a frame rate of 60 Hz and a resolution of 1920 × 1200. Simultaneously, IR images were taken by a VarioCAM HD head 820 S (InfraTec, Germany) at 10 Hz and a resolution of 1024 × 768. The spectral range of the thermal detector is 7.5 to 14.0 µm and, therefore, in the LWIR range. The neonates were recorded in an open incubator.

Since many images From these recordings, 30 images per recording (600 in total) were manually chosen, following three rules:

1. Care was taken not to select images taken immediately after each other to ensure that images of the same recording are not redundant.
2. For each recording, the images were selected to include all body positions, movements, and other activities, such as interaction with the clinician.
3. Since the cameras filmed at different frequencies, the chosen RGB and LWIR images were ensured to depict the same point in time.

Subsequently, the RGB images were labeled using the MATLAB Image Labeler app from The MathWorks, Inc. Opposite to other research [20, 30], where seven classes “background”, “head”, “torso”, “left arm”, “right arm”, “left leg” are common, we did not distinguish between left and right. As described in “Introduction” section, the central–peripheral temperature difference is mostly measured using only two temperature probes. Differentiating between left and right is not standard practice. Hence, we defined the following five classes: “background”, “head”, “torso”, “arm”, “leg”. Moreover, only visible skin was labeled as part of the body part classes, while clothing was labeled as background (see Fig. 8a). In the following, this dataset is referred to as the Chennai dataset.

**Fig. 7:**
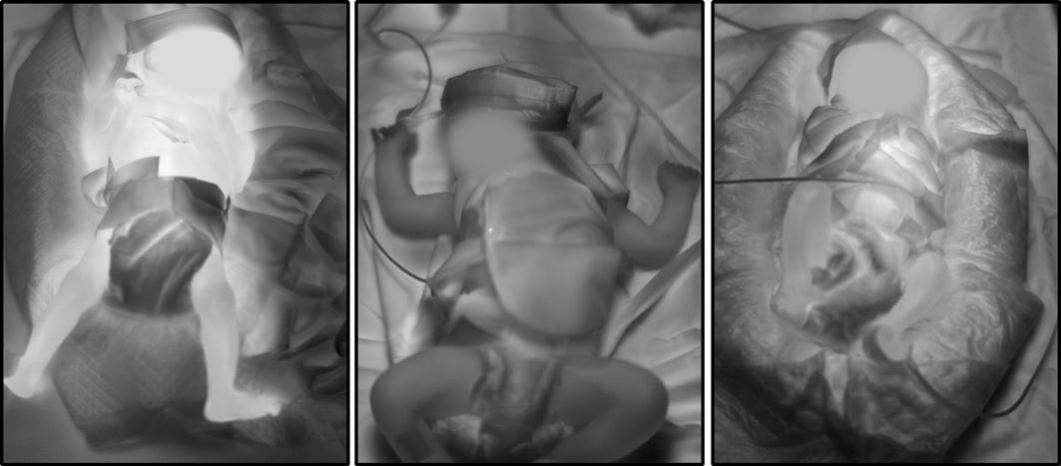
LWIR recordings with different contrasts due to the radiant heater

Since the Chennai dataset only includes images of infants from a specific area in India, the dataset does not cover all possible human skin colors, and trained deep learning models may be biased in this respect. In addition, the radiant heaters of the incubators were turned on during the recordings of seven infants but switched off during the other recordings. Therefore, the contrast between body parts and background in the LWIR images varies between the infants. When the heaters were turned on, the surroundings of the infants were generally warmer than when the heaters were turned off. The warmer skin is thus better distinguishable from the background in the images taken when the heaters were turned off (see Fig. 7). For all thermal images, the gray values were mapped to the individual min. and max. temperature, ensuring a maximum contrast for the dataset.

#### Image registration

As the two cameras have different fields of view (FOVs) and also captured the infants from slightly different angles, an image registration is needed to match both modalities. Furthermore, since the distance and angle of the camera setup varied for each infant, we conducted the image registration for each recording separately. For this purpose, we manually labeled six corresponding points of 5 image pairs, resulting in 30 corresponding points per recording. These points were evenly selected over the entire image so that there is no bias towards a specific area. Since the open incubator and the individual cameras were exchanged or moved for each measurement, we had to find new correspondences for each recording.

**Fig. 8:**
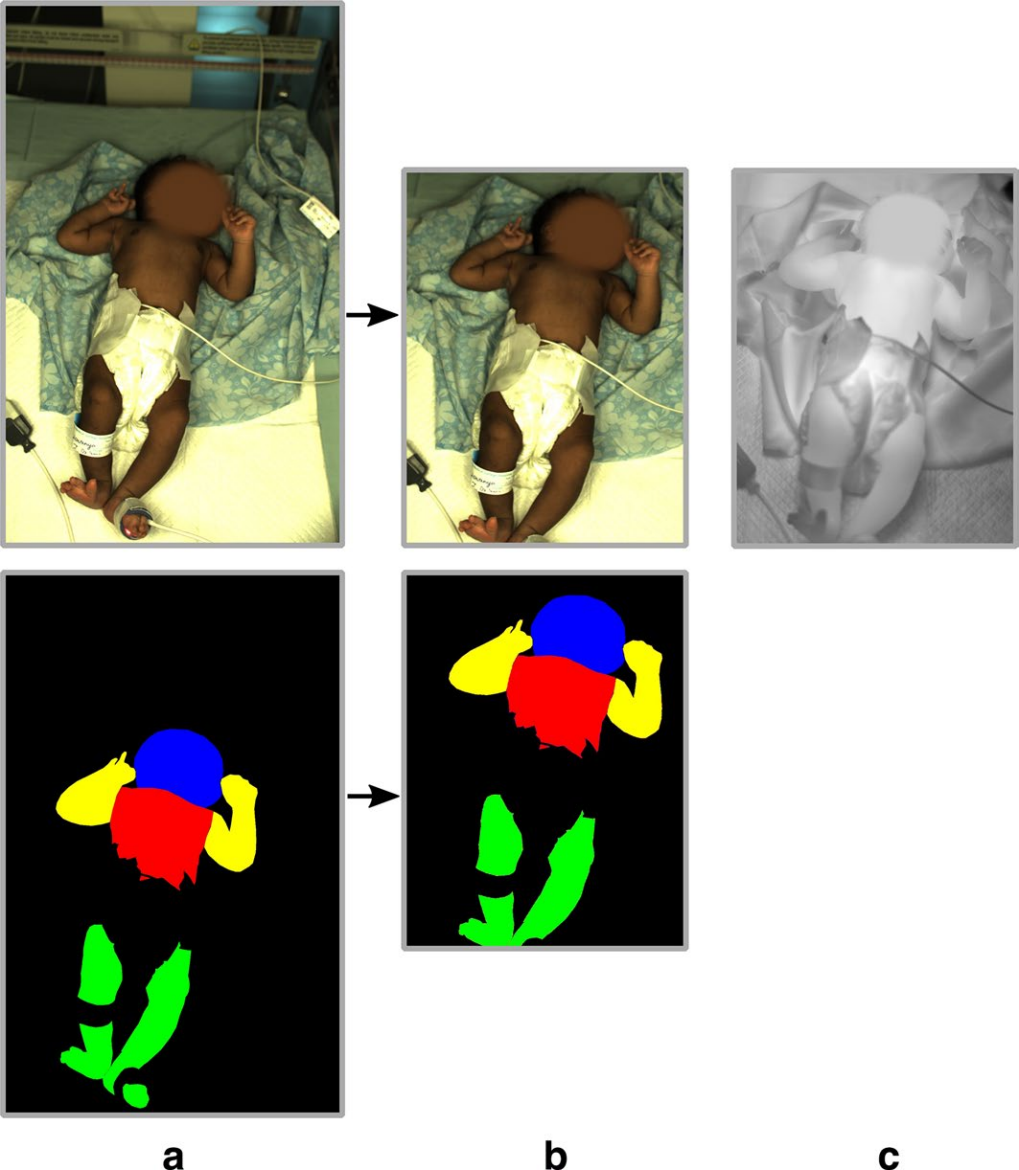
**a** Manually labeled ground truth for the RGB image; **b** transformed RGB image and Ground Truth; **c** corresponding LWIR image

In order to perform the registration of the RGB and Ground Truth image into the LWIR image, we used homogeneous coordinates, as it allows the transformation to be done with a single $3 \times 3$ matrix. Instead of commonly used libraries like OpenCV, we found that applying the L1 loss yielded the best results for the training of the transformation matrix. The L1 loss allows for better acceptance of outlier values inside the image data. However, since the amount of manually labeled training points is rather low, some protection against overfitting is important. That is why we also used the L1 norm for regularization. This allows for a slightly higher variation in the transformation matrix, while at the same time having a regularization effect. An example of the transformation of the images can be seen in Fig. 8.

For the evaluation of the transformation quality, the root-mean-square error (RMSE) for both spatial dimensions between all transformed RGB points and LWIR points of a separate test set was calculated. This resulted in an RMSE of $(X,Y)=(6.3\,\text{px},10.5\,\text{px})$, respectively, ${(X,Y)=(3.15\,\text{mm},5.25\,\text{mm})}$. In contrast, we also tested the OpenCV function for the generation of transformation matrices, which resulted in a worse RMSE of $(X,Y)=(15.6\,\text{px},26.3\,\text{px})$, respectively, $(X,Y)=(7.8\,\text{mm},13.15\,\text{mm})$.

However, the transformations are still improvable. Even though the perspectives of the two cameras only varied slightly during the recordings, small parts of the skin may, for instance, be obscured by clothes when viewed from the first but not from the second perspective. Furthermore, the points from which the transformation matrices are calculated, were selected manually and cannot coincide perfectly. In total, the label masks do not match the transformed LWIR images as well as they match the RGB images they are created from. Thus, the initial conditions for training a network on the LWIR images are worse than for training a network on the RGB images. This needs to be considered when comparing performance on RGB and LWIR images, as well as the fusion of both.

### Network architecture

**Fig. 9:**
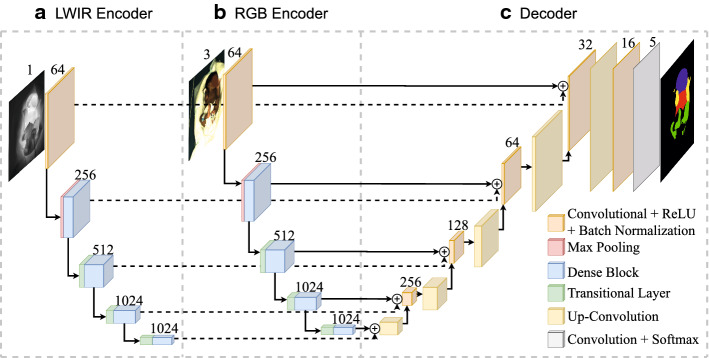
The modified U-Net architecture with **a** an LWIR encoder based on a DenseNet 121; **b** an RGB encoder based on a DenseNet 121; **c** the decoder fusing the outputs of both encoders

Body part segmentation of infants is a semantic segmentation task in the medical field, with limited available data. Therefore, we chose a U-Net architecture in this work, since it was specifically developed for this kind of problem. Furthermore, the U-Net has shown success in a variety of similar semantic segmentation and/or image fusion applications in the past [31]. The proposed network architecture can be seen in Fig. 9.

Based on the U-Net, our neural network has an encoder–decoder structure. While the encoder branch extracts features from the input image, the decoder branch combines the features and outputs the segmentation mask. In addition, the skip connections add high-resolution features to the decoder. In contrast to the unimodal networks (LWIR or RGB images), where only one encoder branch is used, the hybrid fusion network consists of two individual encoder branches. The features of both branches are then fed into the decoder. The fusion is done by pixel-wise addition in order to reduce the number of parameters.

**Fig. 10:**
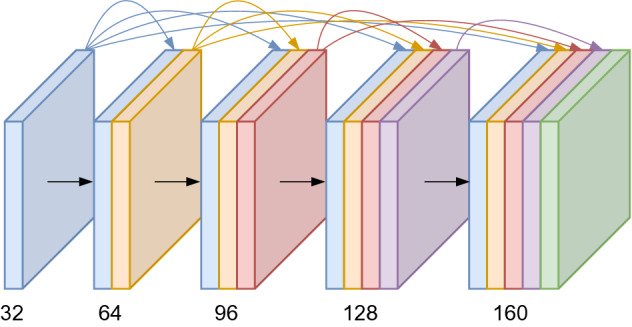
Dense Block with four layers and a growth rate of 32, as proposed by [32]

Our encoder structure is based on a DenseNet-121, proposed by Huang et al. in 2017 [32]. In contrast to the popular ResNet, a DenseNet concatenates the features instead of summing them. The basic structure can be divided into Dense Blocks and Transitional Layers. While the dimensions of the feature maps remain the same within a Dense Block, the number of features changes depending on the growth rate. The Dense Block structure with 4 layers and a growth rate of 32 can be seen in Fig. 10.

Each layer of the Dense Block has six consecutive operations:

- Batch normalization
- Rectifier unit (ReLu)
- 1 × 1 Convolution (reduction to 128 filters)
- Batch normalization
- Rectifier unit (ReLu)
- 3 × 3 Convolution with filter size determined by the growth rate.

The layers between the Dense Blocks are called Transitional Layers and are needed for the downsampling of the dimension and subsequently apply batch normalization, a ReLu activation, a 1 × 1 convolution, and finally, 2 × 2 average pooling.

This architecture has several advantages. The vanishing gradient problem is minimized by connecting every layer directly to the others with a skip connection. Additionally, the skip connection of the layers improves the feature propagation and allows a reduction of total parameters. We used a growth rate of 32 for our encoder, resulting in seven million trainable parameters (14 million parameters for hybrid fusion).

The decoder branch consists of five similar and sequential blocks. Each block contains an up-convolutional layer followed by a 3 × 3 convolution layer, with batch normalization and a ReLu activation being applied after both. The convolution layers have a maximum filter size of 256, which is halved after each block. In the last layer, a softmax activation is applied, which outputs the class probabilities of each pixel. The final segmentation mask is then produced using an argmax function. The decoder structure has an additional seven million parameters, which results in a total of 14 million trainable parameters for the proposed network (21 million for the hybrid fusion network).

#### Implementation

All networks were implemented in Python, using the Keras library. The Adam algorithm proposed by Kingma et al. [33] with the default Keras parameters was chosen as optimizer. Adam complements the gradient descent approach by applying the concept of momentum. This concept aims to avoid converging into a local instead of the global minimum. To achieve this, the weights are updated not only based on the current gradient of the loss function. Instead, the gradients from previous steps are also taken into account when the weights for the next iteration are determined. This way, local minima are more likely to be overcome [34]. Furthermore, we selected the commonly used cross-entropy as loss function.

### Data augmentation

Since the Chennai dataset is relatively small, different augmentations were used to artificially extend it and thus improve the generalization ability of the neural networks. After extensive testing, we chose a combination of five augmentations. The maximum extent to which the augmentations are applied (e.g., the maximum scaling factor or the maximum rotation angle), as well as the probability (*p*) of application are specified for each augmentation separately:

- Images are randomly **rotated** by a maximum rotation angle of 30° (*p* = 0.9). In the task at hand, rotations may be helpful, as a broader range of angular positions of limbs can be covered this way.
- Randomly **scaling** images up or down by a factor between 0.8 and 1.2 (*p* = 0.9) may improve generalization, as the camera may not permanently be mounted at an identical distance from the infant.
- Images are randomly **shifted** horizontally and vertically (*p* = 0.9, relative shift limit = 0.2). Although individual filters in a convolutional neural network are invariant to translation, shifting an image may improve generalization. Since the infants can move, individual body parts may leave the area recorded by the camera. This, in turn, can be simulated by the shift augmentation.
- Horizontal **flipping** of the image (*p* = 0.5), as we do not distinguish between left and right body parts.
- **Coarse dropout** of rectangular regions in the image (max. holes = 12, min. holes = 4, min. height = 80, min. width = 80, max. height = 100, max. width = 100, *p* = 0.8). This method, proposed by DeVries et al., is randomly masking out rectangular regions, which may help to improve the robustness of the neural network [35].

### Transfer learning

In addition to the augmentations, we also applied transfer learning to face the challenges of the small Chennai dataset. As presented by Hoog Antink et al., the accuracy of infant segmentation improves when a neural network is pre-trained on data of adult persons [20].

In a first step, we used weights for the encoder pre-trained on the ImageNet dataset. It consists of 1,461,406 manually annotated images classified into 1000 different object classes [36]. This dataset was initially made for image classification rather than semantic segmentation. Nevertheless, neural networks for segmentation tasks often use weights pre-trained on the ImageNet dataset due to its large size and because the learned kernels can be transferred [31, 37].

**Fig. 11:**
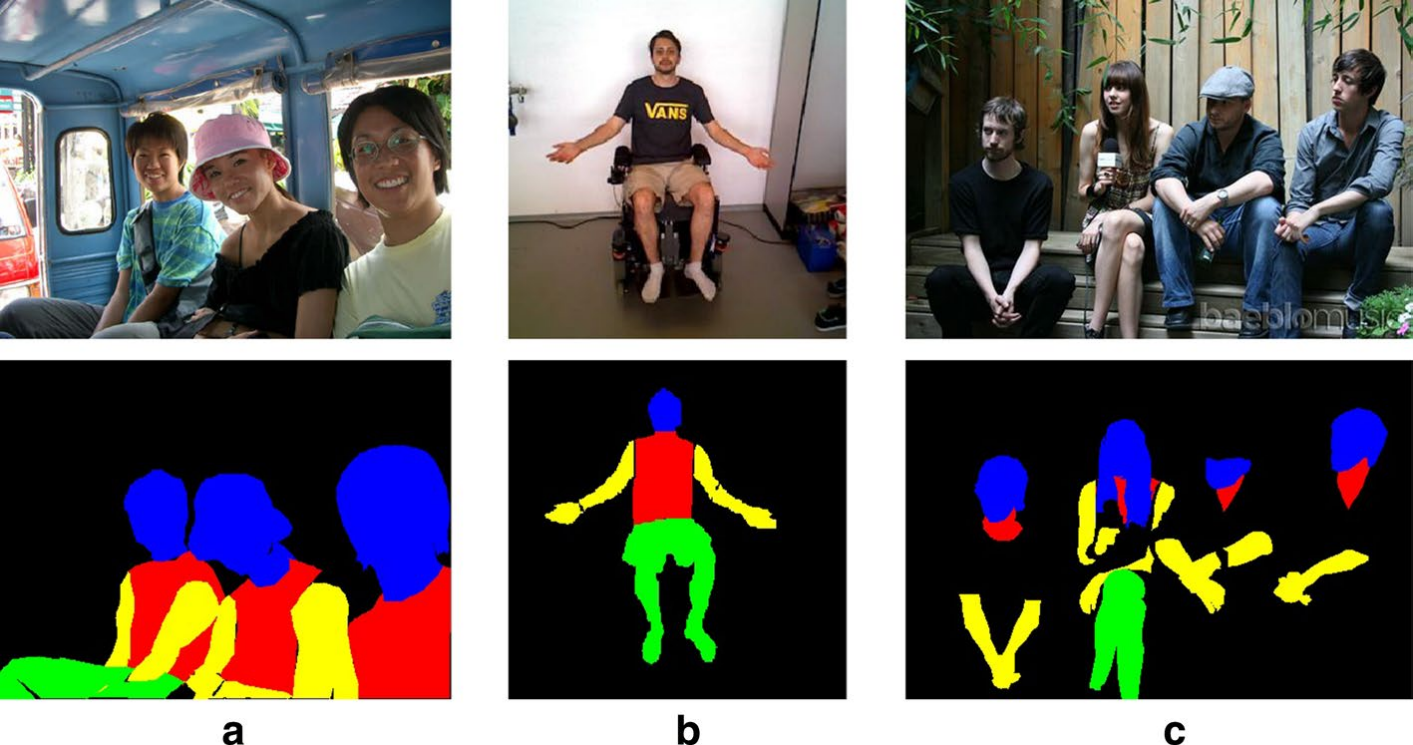
Examples from **a** Pascal-Person-Part Dataset [38]; **b** Freiburg Sitting People dataset [30]; **c** Crowd Instance-level Human Parsing dataset [40]

In a next step, we used datasets of adults to pre-train our network for the task of body part segmentation. Hoog Antink et al. used a combination of the Freiburg Sitting People dataset [30] and the Pascal-Person-Part dataset [38], which is a subset of the VOC 2010 dataset [39]. In the following, this combined dataset, consisting of 3733 manually labeled images, is only referred to as the Pascal–Freiburg dataset. Additionally, we used the Crowd Instance-level Human Parsing (CIHP) dataset for pre-training [40]. This dataset contains 38,280 manually labeled images, making it more than ten times bigger than the Pascal–Freiburg dataset. We merged the classes into head, torso, arm, leg, and background for both datasets, matching the classes of the Chennai dataset. In contrast to the Chennai and CIHP dataset, the Pascal–Freiburg dataset does not distinguish between visible skin and body parts with clothes. However, the infants of the Chennai dataset mostly do not wear clothes on the legs and arms. Therefore, the difference between the “leg skin” and the “leg skin and clothes” class may be slight. Only the torso is often covered by clothes, which leads to a significant difference between the “torso” and the “torso skin and clothes” classes. Nevertheless, since transfer learning is to be applied, the classes used for pre-training do not need to coincide with the classes eventually aimed at. An example for both adult datasets, including their ground truths, is shown Fig. 11.

As the adult datasets only contain RGB images, we transformed the datasets to a grayscale format for the pre-training of the LWIR model since no annotated dataset of LWIR images of adults is publicly available.

#### Fusion pre-training

We compared different pre-training strategies to optimize the training of the fusion network. The evident approach is pre-training the encoder and decoder weights on the adult dataset. However, since we use grayscale images for pre-training the LWIR encoder, this might not be optimal. By using the best weights from the unimodal networks, it might be possible to optimize the two encoder branches and the decoder. In total, four different approaches were compared:

1. Select encoder and decoder weights from pre-training on the adult dataset.
2. Select the best encoder weights of the unimodal networks and decoder weights from pre-training on the adult dataset.
3. Select the best encoder weights of the unimodal networks and the best decoder weights of the LWIR network.
4. Select the best encoder weights of the unimodal networks and the best decoder weights of the RGB network.

### Evaluation method

As the Chennai dataset is comparatively small, we evaluated our neural network using a fivefold cross-validation. The 600 RGB and 600 LWIR images were split into fivefolds. In addition, an independent test set for final evaluation was created. Each fold contains 90 RGB and 90 IR images, while the test set contains the remaining 150 RGB and IR images. An overview of the data split is shown in Table 2. All images of one recording were assigned to only one fold. As a result, five complete recordings were left out for final evaluation with the test set.

**Table 2:** Split of the Chennai dataset

Fold	1	2	3	4	5	Test
Number of recordings	3	3	3	3	3	5
Total image pairs	90	90	90	90	90	150

One exception to this split is fold 2 and fold 4, which have two separate recordings of one infant. However, since the two recordings were taken on different days with different clothing and backgrounds, we have assigned them to separate folds.

The tuning of hyperparameters, such as augmentation, network architecture, and pre-training, was performed exclusively during cross-validation. The final models were evaluated only once on the test data set at the end. This was done to avoid the peeking effect, where iterative revision of the models causes the hyperparameters to eventually be tuned on the test data [41].

The widely used metric Intersection-over-Union was selected for the comparison of the different neural networks. This metric compares the area assigned to class *c* by the ground truth mask ($y_{\text{gtruth},c}$) to the area predicted by a neural network ($y_{\text{prediction},c}$). This is done by dividing their intersection by their union:

1 $${\text{IoU}}_{c} = \frac{|y_{\text{gtruth},c} \cap y_{\text{prediction},c} |}{|y_{\text{gtruth},c} \cup y_{\text{prediction},c} |}.$$

We note that the IoU can have values between 0 (no overlap) and 1 (both are identical). Furthermore, the mean IoU (mIoU) indicates accuracy averaged over the four classes head, torso, arms, legs, leaving out the background class.

## Data Availability

The presented database of infants cannot be made publicly available, due to the declarations in the ethics proposal.
